# Physiologists as medical scientists: An early warning from the German academic system

**DOI:** 10.14814/phy2.70055

**Published:** 2024-10-27

**Authors:** Katrin Streckfuss‐Bömeke, Nicolle Kränkel, Christoph Maack, Renate B. Schnabel, Laura C. Zelarayán, Norbert Frey, Peter Jezzard, Martina Krüger, Nico Lachmann, Susanne Lutz, Claudia Noack, Eric Schoger, Katrin Schröder, Laura C. Sommerfeld, Sabine Steffens, Holger Winkels, Christina Würtz, Tanja Zeller, Eva A. Rog‐Zielinska, Peter Kohl

**Affiliations:** ^1^ Institute of Pharmacology and Toxicology, University of Würzburg Würzburg Germany; ^2^ Clinic for Cardiology and Pneumology Georg‐August University Göttingen Göttingen Germany; ^3^ DZHK (German Center for Cardiovascular Research), Partner Site Göttingen Göttingen Germany; ^4^ Deutsches Herzzentrum der Charité, Klinik für Kardiologie, Angiologie Und Intensivmedizin Campus Benjamin‐Franklin (CBF) Berlin Germany; ^5^ DZHK (German Centre for Cardiovascular Research) Partner Site Berlin Berlin Germany; ^6^ Friede Springer Centre of Cardiovascular Prevention at Charité Charité—University Medicine Berlin Berlin Germany; ^7^ Comprehensive Heart Failure Center University Clinic Würzburg Würzburg Germany; ^8^ Medical Clinic 1 University Clinic Würzburg Würzburg Germany; ^9^ Department of Cardiology University Heart & Vascular Center Hamburg, University Medical Center Hamburg‐Eppendorf Hamburg Germany; ^10^ German Center for Cardiovascular Research (DZHK) Partner Site Hamburg/Kiel/Lübeck Hamburg Germany; ^11^ Institute of Pharmacology and Toxicology, University Medical Center Göttingen Germany; ^12^ Medical Clinic I, Cardiology and Angiology, Experimental Cardiology Justus‐Liebig‐University Giessen Germany; ^13^ Department of Medicine III: Cardiology, Angiology, and Pneumology Heidelberg University Heidelberg Germany; ^14^ DZHK (German Centre for Cardiovascular Research), Partner Site Heidelberg Germany; ^15^ Wellcome Centre for Integrative Neuroimaging University of Oxford Oxford UK; ^16^ Institute of Cardiovascular Physiology, University Hospital Düsseldorf Düsseldorf Germany; ^17^ Cardiovascular Research Institute Düsseldorf Düsseldorf Germany; ^18^ Department for Pediatric Pneumology, Allergology and Neonatology Hannover Medical School Hanover Germany; ^19^ German Center for Lung Research (DZL) Biomedical Research in Endstage and Obstructive Lung Disease Hannover (BREATH) Hanover Germany; ^20^ Cluster of Excellence RESIST (EXC 2155) Hannover Medical School Hanover Germany; ^21^ Fraunhofer Institute for Toxicology and Experimental Medicine Hanover Germany; ^22^ Nuvisan ICB GmbH, Department Therapeutic Research Berlin Germany; ^23^ Institute for Cardiovascular Physiology, Goethe University Frankfurt Am Main Germany; ^24^ DZHK (German Centre for Cardiovascular Research), Partner Site RheinMain Frankfurt Germany; ^25^ University Center of Cardiovascular Science (UCCS), University Heart and Vascular Center Hamburg, University Medical Center Hamburg‐Eppendorf Hamburg Germany; ^26^ Institute for Cardiovascular Prevention (IPEK), Ludwig‐Maximilians‐Universität Munich Germany; ^27^ DZHK (German Centre for Cardiovascular Research), Partner Site Munich Heart Alliance Munich Germany; ^28^ University of Cologne, Faculty of Medicine and University Hospital Cologne, Clinic III for Internal Medicine Cologne Germany; ^29^ Center for Molecular Medicine Cologne University of Cologne Cologne Germany; ^30^ Institute of Cardiovascular Physiology University Medical Center Göttingen Göttingen Germany; ^31^ Office of the Dean of Studies Faculty of Medicine/University Medical Center Göttingen Göttingen Germany; ^32^ University Center for Cardiovascular Science University Heart & Vascular Center Hamburg Hamburg Germany; ^33^ Institute for Experimental Cardiovascular Medicine University Heart Center Freiburg · Bad Krozingen, University of Freiburg Freiburg Germany; ^34^ Faculty of Medicine University of Freiburg Freiburg Germany; ^35^ CIBSS Centre for Integrative Biological Signalling Studies University of Freiburg Freiburg Germany

**Keywords:** funding, medical scientists, physiologists, professional development, training, white paper

## Abstract

“Medical scientists” are postgraduate investigators who are engaged in biomedical research, and either hold a biomedical PhD or are qualified in medicine but do not participate in patient care. Medical scientists constitute ~40% of staff at medical faculties and >90% at nonuniversity medical research institutions in Germany. However, medical scientists in Germany face limited long‐term career prospects and a lack of dedicated training and support programmes. They also face time limits on their career progression arising from national academic employment legislation, and imminent reforms by the German government are likely to make this worse. Nevertheless, recent developments in the educational landscape including the introduction of increasingly focused MSc, pre‐PhD, and doctoral programmes to train medically aware basic scientists, as well as improved general recognition of the roles and relevance of medical scientists in health research, are encouraging. Physiologists have taken essential steps to improve the recognition of medical scientists in Germany by introducing a “specialist physiologist” qualification; this initiative could be applied to support medical scientists in other fields and countries. In this review, we describe the particular challenges facing medical scientists in Germany and make recommendations that may apply to other academic systems.

## 1 MEDICAL SCIENTISTS

Medical scientists (in some academic communities also referred to as biomedical scientists) are postgraduate investigators qualified in a science, technology, engineering, or mathematics (STEM) subject or in medicine, who are engaged in health research, but who do not participate in patient care. In comparison, clinician scientists are medically qualified, conduct biomedical research, and do treat patients (Table 1). Medical scientists provide diverse perspectives and expertise and are thus indispensable for translational research (Forum Gesundheitsforschung, 2020). However, the concept of “medical scientists” remains poorly recognised, and many physiologists may not even realise that they are medical scientists. Moreover, medical scientists face specific demands in terms of training, career prospects, recognition, and support, all in the context of an increasingly challenging shortage of skilled, medically aware basic scientists in Germany and beyond (European Commission, 2020, 2023; Langin, 2022).

**TABLE 1:** Distinguishing between clinician scientists and medical scientists.

Main qualification	Medicine		STEM subject
Engaged in biomedical research	Yes	Yes	Yes
Treats patients	Yes	No	No
Terminology used	Clinician Scientist	Medical Scientist	

*Note*: Medical scientists are qualified in a science, technology, engineering, or mathematics (STEM) subject or medicine, conduct biomedical research, but are not involved in treating patients. Clinician scientists are medically qualified, conduct biomedical research, and they also treat patients. Medically qualified researchers would be considered medical scientists according to this terminology if they are not involved in clinical duties (Forum Gesundheitsforschung, 2020).

The German Cardiac Society (DGK) and the German Centre for Cardiovascular Research (DZHK) held a Translational Workshop in Bonn, Germany, in October 2023 to explore this complex topic. This paper is based on the findings of the workshop and reflects on the current status of medical scientists, with a particular focus on the example of cardiovascular research in Germany.

## 2 CLINICIAN SCIENTISTS: A SUCCESS STORY

A key challenge for clinician scientists is that they lack time for research while fulfilling their clinical duties. Until recently, many clinicians could only perform laboratory‐based research during the early stages of their professional development, or while “off duty” later on.

Recognising the cost of losing out on research by highly motivated physicians, as of 2022 nearly all 39 medical faculties at German public universities had implemented (or were in the process of implementing) clinician scientist support schemes that offer “buyouts” from clinical duties and a curriculum of cross‐disciplinary training (Stiftung Charité, 2023; Pittet, 2023; Medizinischer Fakultätentag, n.d..; Medizinischer Fakultätentag, 2020). So, could the support programmes available to clinician scientists serve as a blueprint for medical scientist support?

## 3 MEDICAL SCIENTISTS: THE STATE OF PLAY

**FIGURE 1:**
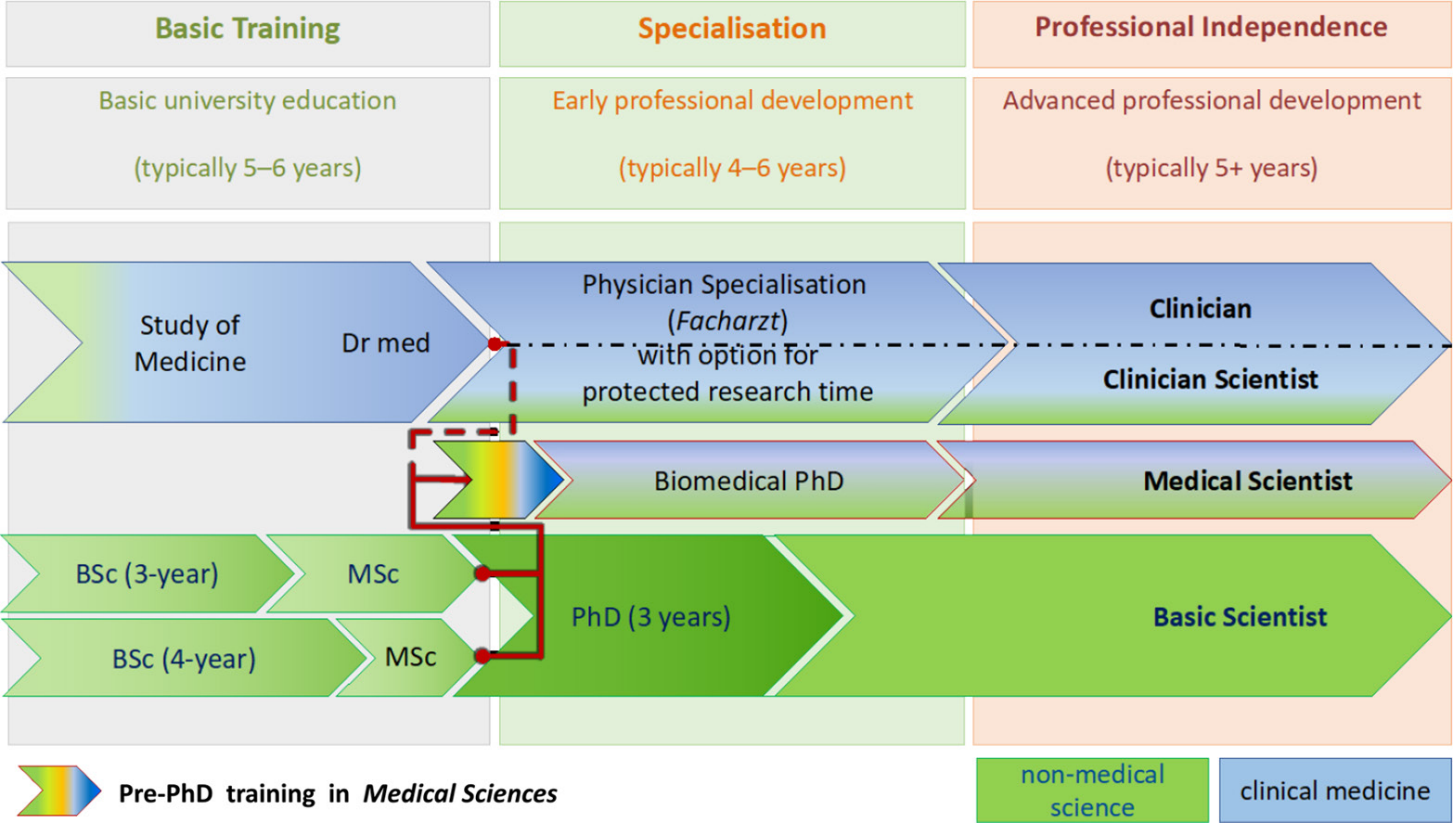
Training and education for basic scientists, medical scientists, clinicians, and clinician scientists. Top row of arrows: Basic university education (left) for physicians can involve thesis work so that at the start of the specialisation period (middle), clinician scientists usually hold a medical doctorate degree. Research during this period (leading to a Certificate of Specialist Training (*Facharzt*)), and during the subsequent development of professional independence (right), can be supported through early and advanced clinician scientist programmes. Bottom row of arrows: Basic scientists complete BSc/MSc programmes at university before they enter PhD training during the specialisation period, and before pursuing professional independence as a postdoc. Middle row of arrows: Medical scientists need to also acquire an understanding of medical research needs. This can occur before, during, or after PhD research.

Medical scientists and clinician scientists go through comparable stages of training (Figure 1), which we refer to as *basic training* (5–6 years of BSc, MSc, MD, or equivalent studies); *specialisation* (roughly 4–5 years for a PhD in an STEM subject (Jaksztat et al., 2012) or medical specialisation); and *professional independence* (3–4 years post PhD or specialisation). However, medical scientists face their own set of challenges.

*Precarious career prospects*: Although specific numbers for physiologists are unavailable, data on physicians and non‐physicians suggest that similar proportions of clinician scientists and medical scientists have permanent positions at German medical faculties (approximately 44% of physicians and 45% of nonphysicians; Forum Gesundheitsforschung, 2020). However, the situation for the 55% on non‐permanent contracts differs, as many medical scientists (i.e., those without medical training) will not be able to “fall back” on practising medicine if their career in research does not work out.

Indeed, data suggest that most doctoral graduates struggle to progress in their academic careers in Germany regardless of discipline. In 2023, just 1592 scientists completed a habilitation (a postdoctoral qualification that is seen as a formal requirement for professorship in the German academic system) and just 37% of scientists who habilitated were women (Destatis Statisches Bundesamt, 2024).

Professorships at German medical faculties are also distributed unevenly depending on professional background. A 2017 survey suggested that 8.1% of medically trained staff held a professorial title at German university medical faculties, compared to just 4.1% of nonmedically trained staff. At nonuniversity medical research institutions, nearly a quarter (24.1%) of medically trained staff held a professorship, compared to just 3.4% of nonmedically trained staff (Forum Gesundheitsforschung, 2020). Assuming that most medical scientists have not had medical training, these data suggest that medical scientists are less likely to achieve the rank of professor than their clinician or clinician scientist colleagues.

A contributing factor is the relative scarcity of professorships: across all subjects, there were 200,300 students undertaking doctoral studies and approximately 50,000 professors in Germany in 2021 (a 4:1 ratio (Statista (a), n.d.; Destatis Statisches Bundesamt, 2022)). When accounting for the substantially longer tenures of professorial appointments compared to the typical duration of doctoral studies (say, 25 years vs. ~4.5 years), the realistic chances of any particular doctoral student in Germany eventually attaining a professorship may be roughly 1 in 20. The situation in Germany appears less favourable than in Sweden or the Netherlands, where the ratios of students to professors are around 3 to 1 (Statista (b), n.d..; Silander & Pietilä, 2023; de Goede et al., 2013).

The fact that very few PhD students will become professors needs to be communicated more proactively to trainees. The Coalition for Next Generation Life Sciences is an example of a group of (predominantly North American) universities and research institutions that collect and publish data on the career outcomes of their graduates (https://nglscoalition.org). Encouragingly, funding bodies in Germany and Europe have started to collect similar information.

*Women are more likely to leave academia*: Worryingly, female researchers continue to be more likely to lose out on a career in academia compared to their male counterparts across all disciplines. Although the proportion of female and male doctoral students in Germany is reasonably well balanced (48% vs. 52%, respectively (Destatis Statistiches Bundesamt, 2023a)), only 37% of habilitations were awarded to women in 2023 and just 28% of 51,200 full‐time professorships were held by women in 2022 (Destatis Statistiches Bundesamt, 2023b; Destatis Statistiches Bundesamt, 2024; GWK, 2023). A report by the DGK suggests that this mirrors trends seen in cardiology, where just 3.4% of those working at the director level are women (Lerchenmüller et al., 2023), and national statistics that place Germany near the bottom of European Union in terms of the share of women working in research overall (29.4% in 2021) (Destatis Statistiches Bundesamt, 2023c). This trend is not limited to Germany: in the USA, women are underrepresented among faculty in nearly all academic fields, and they are more likely to leave academia at every career stage, frequently feeling “pushed out” due to workplace climate (Spoon et al., 2023). Similarly, female life science researchers in the UK are less likely to progress in their careers or to remain in academia than their male counterparts (Dias Lopes & Wakeling, 2022). These figures indicate troubling structural problems that disproportionally affect women internationally.

*Limited structured support*: Currently, medical scientist support programmes lag behind the multi‐tiered training and support structures for clinician scientists in Germany. The DFG (the leading German federal funding body for research) and the German Federal Ministry of Education and Research (or BMBF) support 23 early clinician scientist and 8 advanced clinician scientist programmes across Germany (Bundesministerium für Bildung und Forschung, n.d.‐a; Deutsche Forschungsgemeinschaft, n.d.)—but not one programme that is specifically focused on medical scientists. To date, the Else Kröner‐Fresenius Stiftung (EKFS) offers the only national funding scheme dedicated to supporting structured training for medical scientists (Else Kröner Fresenius Stiftung, n.d.). As of 2023, 6 EKFS schools for Medical Scientists exist, each supported with €1.1 million of funding for a 4‐year term (Else Kröner Fresenius Stiftung, n.d.).

A number of professional bodies have expressed concerns over levels of funding and support for physiological research internationally (Gregorio, 2019; Rodrigues et al., 2024; Sengupta & Barman, 2018). Like clinician scientists, medical scientists of all disciplines are eligible for highly competitive personal fellowships, such as those offered by the DFG (Deutsche Forschungsgemeinschaft, 2022). The most advanced levels of these fellowships require a commitment by the host faculties to turn supported positions into tenured posts upon completion, which is becoming increasingly difficult (if not backed up by a “professional position,” such as for physicians in university hospitals).

With their host institution's permission, medical scientists can also independently apply for “standard research grants” that include their salary, but these are generally not tailored to the requirements of medical scientists. Funding bodies may expect recipients to dedicate 100% of their time to grant‐funded research, precluding other important postdoctoral career development activities such as teaching, academic self‐governance, or structured network initiatives. Here, programmes offering buyouts from grant‐funded research could foster broader academic engagement and networking. This would illustrate commitment on the part of host institutions and (as only part of the principal investigator's salary would have to be sought externally) could increase flexibility in funding requests or even increase funding rates, particularly for medical scientists applying for their first independent research grant.

*Time limits on career progression*: A major challenge for medical scientists in German academia is the strict time limit imposed on their professional development by the “Wissenschaftszeitvertragsgesetz” (the “Act on Fixed‐Term Employment Contracts in Academia” or *WissZeitVG*), which, at the time of writing, will soon be updated to restrict nonpermanent employment on intramural funding to just 4 years after obtaining a PhD (Bundesministerium für Bildung und Forschung, 2020; Davidson et al., 2023; Bundesministerium für Bildung und Forschung, n.d.‐b). This is in spite of data showing that medical scientists require, on average, 8.6 years from completion of their PhD to attain permanent posts in academia—more than twice the limit allowed in the new proposals (Kordel et al., 2022). While the *WissZeitVG* includes family‐related provisions (allowing an extension to the time limit of 2 years per child for academics with children), the clock does not reset if a researcher changes university, unlike in other countries with similar constraints on duration of employment (Davidson et al., 2023).

While the updated law is intended to accelerate scientists' career progression and enable a better work–life balance, it will likely worsen the situation for early career medical scientists by increasing workloads, pressure to publish, and competition for already scarce permanent positions, while also reducing the time available for gaining essential independent expertise in research and teaching. As the updated law is not linked to an increase in university funding, it may contribute to a “brain drain,” as researchers leave Germany, academia, or science altogether—and paradoxically cause researchers to delay wider life goals, and thereby negate the intended improvements to work–life balance (Consortium for the National Report on Junior Scholars, 2021; Zelarayán et al., 2023). Perhaps reflecting these pressures, the proportion of PhD students in Germany who wish to work in academia fell from 22% in 2017/2018 to 14% in 2021/2022, and up to 80% of postdoctoral researchers will leave academic research and teaching to work in other sectors (Deutsches Zentrum für Hochschul‐ und Wissenschaftsforschung (DZHW), 2024; Deutsches Zentrum für Hochschul‐ und Wissenschaftsforschung (DZHW), 2014).

Academic employment law in Germany is clearly at odds with reality and will likely continue to undermine medical scientists' career prospects.

*Medical scientist careers—all or nothing*? These challenges contribute to a feeling that the occupation of “medical scientist” is not a “profession” in the classic sense but a “career,” as medical scientists must demonstrate continual and unrealistically rapid professional progression through ever‐higher ranks in the professional hierarchy if they are to finally obtain secure, permanent employment. As a full professorship is not a realistic prospect for most early career medical scientists, new ideas are needed to safeguard long‐term prospects for medical scientists, both within and beyond academia. However, this is complicated by a lack of professional representation of medical scientists' interests in the broader scientific community.

## 4 BUILDING A MEDICAL SCIENTIST BRAND

Recognition and representation of physiologists and medical scientists remains limited, particularly within Europe (Eisner et al., 2013; Rodrigues et al., 2024). Awareness could be improved by building a medical scientist “brand” that represents the common interests of medical scientists. In this regard, physiologists have made important progress by introducing the “specialist physiologist” (*Fachphysiologe*) title. This title was established by the German Physiological Society (DPG) and is awarded to qualified scientists working in the field of physiology after obtaining relevant qualifications and experience in research, publications, teaching, and didactics over a 5‐year period (usually overlapping with PhD training (Deutsche Physiologische Gesellschaft, n.d.)). The *Fachphysiologe* title is intended to demonstrate the medical scientist's ability to perform independent scientific research and training in the field of physiology, with the goal of improving future employability.

A similar specialisation programme and certificate for physiologists and other medical scientists working in cardiovascular research, under the auspices of national bodies like the DGK and DZHK, could establish a brand (e.g., *Medical Scientist—Cardiovascular Research*), raise awareness, and aid training and professional development of medical scientists. A first step could be the development of a common postgraduate training curriculum that can be shared across emerging medical scientist programmes to ensure they are consistent and coherent, and to help build a sense of identity, common purpose, mobility, and cohesion among medical scientists. This could mimic the DGK Academy's continuing medical education (CME)‐certified courses for physicians (though tailored to medical scientists).

The various subject‐specific professional societies that offer dedicated training for medical scientists, such as the DPG, DZHK, and DGK, could also work together to support medical scientist course development, improve networking and lobbying, and perhaps develop accreditation criteria for new medical scientist training programmes.

Regardless, beyond the need for a medical scientist “brand,” there is a broader need for structured training and support for medical scientists.

## 5 MEDICAL SCIENTISTS: TRAINING AND SUPPORT

*Terminology and concepts*: It would not be prudent to copy clinician scientist training and support structures for medical scientists on a one‐for‐one basis, as their key needs differ.

This begins with exactly when researchers qualify for what is typically “postdoctoral” support.

As most clinician scientists in the German system conduct medical doctorate research projects in parallel with their undergraduate education, they therefore generally qualify for postdoctoral support upon completion of their undergraduate education, at an average age of 25.9 years (Statista (c), n.d.). For medical scientists, doctoral research commences *after* completion of BSc and MSc studies, and the average PhD takes 4.7 years (Consortium for the National Report on Junior Scholars, 2021). Medical scientists consequently qualify for postdoctoral support at an average age of 31.7 years, 6 years later than clinician scientists (Kordel et al., 2022). What is more, “postdoctoral” support would become available to medical scientists after, not during, the critical phase of their professional training and specialisation. If support programmes for medical scientists are to match the opportunities available to early and advanced clinician scientists in spirit and not merely in name, they should provide support for early (i.e., during or even before PhD studies) and advanced medical scientists (after completion of their research‐based doctorate; Figure 1).

*Examples of training programmes*: Current training programmes for medical scientists can be broadly divided into *basic training*, *specialisation*, and development toward *professional independence* (Figure 1). *Basic training* programmes for nonclinicians in cardiovascular research are scarce; they include the University Medical Centre Göttingen's 2‐year MSc in *Cardiovascular Science*, and the University of Freiburg's 1‐year “pre‐PhD” in *Medical Sciences—Cardiovascular Research*. Both introduce STEM scientists to a specific facet of medical research—in this case, the cardiovascular system—a precondition for fruitful doctoral research.

While *specialisation* training programmes in cardiovascular research are included as dedicated tracks in many graduate schools, or at the heart of dedicated programmes such as the PhD in *Cardiovascular Science* at the University of Göttingen, training and support programmes for postdoctoral medical scientists working toward *professional independence* are also limited at present. They include the Hannover Medical School's Medical Scientist Programme and the University of Freiburg's Hans A. Krebs Medical Scientist Programme. Further details of all these programmes can be found in Appendix A.

## 6 MEDICAL SCIENTISTS: BROADER CAREER PROSPECTS

**FIGURE 2:**
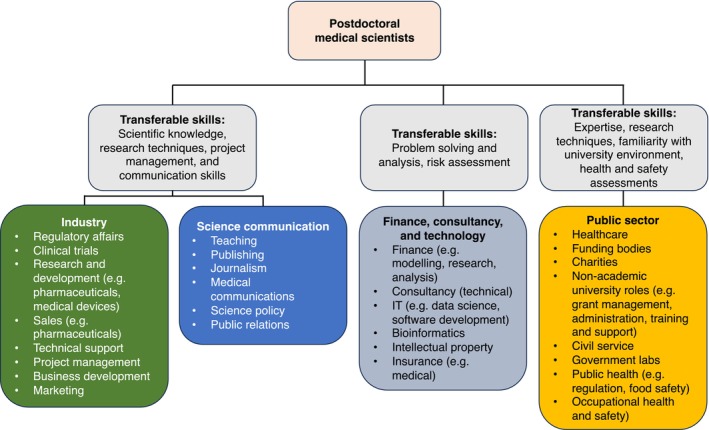
Some of the career options outside of academia and the relevant transferable skills postdoctoral medical scientists may seek to develop.

As medical scientists, physiologists possess valuable subject‐related knowledge and transferable skills, and medical scientists have a wide range of career options outside of academic research (Figure 2). However, medical scientists are typically not well prepared for careers outside academia, due to limited exposure to other career options during training and the negative connotations that are sometimes associated with such a move. Remedying this requires the provision of information relating to career prospects and trajectories (as championed by the Coalition for Next Generation Life Sciences), as well as structural improvements, such as incorporating trainee rotations in industry, science communication, or the public sector into academic curricula. This is an objective of the European Union's Marie Skłodowska‐Curie training networks, which in 2022 funded 149 doctoral programmes, including 14 industrial doctoral programmes to train PhD candidates outside academia (Marie Skłodowska‐Curie Actions, n.d.). However, given the relatively short duration of PhD studies in Germany and the limited time available for postdoctoral development, the incorporation of additional content to existing doctoral training should be carefully considered – or incorporated into pre‐PhD training (such as the Freiburg model mentioned above and described in Appendix A).

## 7 CONCLUSION

Medical research, education, and innovation depend on the interdisciplinary exchange between experts from various backgrounds, including medical scientists, who constitute a sizable fraction of staff in university and nonuniversity health research institutions. It is time that we adjusted our professional and societal approach to training and supporting medical scientists in order to offer the necessary means for protecting and nurturing this category of research staff in Germany and elsewhere. While progress is being made, much remains to be done to improve the standing, training, and support available for physiologists and other medical scientists. A summary of some actions that professional societies, funding bodies, and universities can carry out to improve support for medical scientists working in the field of cardiovascular research in Germany can be found in Appendix B.

Awareness and representation of medical scientists must be improved through education and the involvement of professional bodies like the DGK and DZHK. Financial support and specialist training for medical scientists should begin early, ideally during their PhD studies. Above all, there is a need to improve the long‐term career prospects for physiologists and medical scientists in general, with a focus on female scientists. In particular, the predictably negative effects that time restrictions such as those imposed by the German *WissZeitVG* have on medical scientists' careers must be remedied. Overall, more must be done to ensure that medical scientists have a reasonable chance for a stable, long‐term career, within and beyond academia.

## AUTHOR CONTRIBUTIONS

This paper was written based on discussions at the DGK/DZHK Translational Workshop “Medical Scientists in Cardiovascular Research: contents, structures, challenges, needs” held in Bonn, Germany, on October 4, 2023. The authors contributed to workshop discussions, made suggestions on the manuscript drafted by PK, NK, CM, RBS, KS‐B, and LCZ, and all authors approved the final version.

## FUNDING INFORMATION

The workshop was financially supported by the DGK and the University Heart Centre Freiburg · Bad Krozingen.

## CONFLICT OF INTEREST STATEMENT

KS‐B received research support from Novartis and BionTECH and speaker's honoraria from Novartis. CM is an advisor to Amgen, AstraZeneca, Boehringer Ingelheim, Bristol Myers Squibb, Novo Nordisk, and Servier and received speaker honoraria from AstraZeneca, Bayer, Bristol Myers Squibb, Boehringer Ingelheim, Berlin Chemie, Edwards, Novartis, Novo Nordisk, and Servier. RBS has received lecture fees and advisory board fees from BMS/Pfizer and Bayer outside this work. CN is an employee at Nuvisan ICB GmbH. SSt received a speaker honorarium from NovoNordisk. TZ received funding from Vifor Pharma, is listed as co‐inventor of an international patent on the use of a computing device to estimate the probability of myocardial infarction (International Publication Number WO2022043229A1), and is shareholder of the ART.EMIS GmbH Hamburg. NL participated in the translational workshop as the spokesperson of the nextGENERATION Medical Scientist programme, which is funded by the Else Kröner Fresenius Stiftung. PK is course director of the Freiburg Medical Scientist MSc programme. All other authors have no competing interests to declare.

## ETHICS STATEMENT

This review article did not involve any data collection from human participants or animals. Therefore, no ethics approval was required.

## Data Availability

Data sharing is not applicable to this article as no data sets were generated or analyzed during the current study.

## APPENDIX A Examples of training programmes for cardiovascular medical scientists in Germany

### A.1

Current training programmes for medical scientists can be broadly divided into *basic training*, *specialisation*, and development toward *professional independence*.

*Basic training*: The University Medical Centre Göttingen's 2‐year MSc in Cardiovascular Science is an excellent example of basic training for medical scientists. This English‐language course, running since 2015, is highly selective (with approximately 600 international applications for 25 places) and involves training in basic and clinical cardiovascular science, including cardiac physiology, with a focus on lectures and practical classes. The final semester of the programme is dedicated to master's thesis research undertaken at Göttingen or elsewhere. Approximately 50% of graduates subsequently enter a PhD programme in cardiovascular science, and about half of those do so at Göttingen.

The University of Freiburg's 1‐year “pre‐PhD” MSc in Medical Sciences—Cardiovascular Research, while also including a focus on physiology, takes a slightly different approach. This English‐language course, launched in 2023, offers an interdisciplinary programme with highly interactive seminars and practical instruction in a small group setting of up to 6 STEM subject graduate students per year. The first half‐year term provides an overview of cardiovascular science, from a thorough review of cardiovascular physiology and anatomy to shadowing of clinical interventions, dissection courses, and live cardiac tissue studies, while the second term is dedicated to a master's research project, designed to support subsequent transition to PhD work. Admission is highly selective (with around 60 applications for four places in the first year) and targets trainees who have already decided to pursue a PhD in cardiovascular medicine. The stipend‐supported MSc therefore serves as a pre‐PhD, providing future doctoral students with a broad but deep foundation for their subsequent doctoral research.

Crucially, both programmes provide opportunities to establish an early career network of peer interactions to help build scientific independence and explore potential PhD host labs. The targeted and structured introduction to the medical dimension of a research subject prior to PhD work has multiple benefits, aiding professional development and acquisition of skills and understanding, broadening horizons, and preparing students for professional progress upon completion of their PhD.

*Specialisation*: Many graduate schools in Germany offer subject‐specific training programmes for PhD students. The University of Göttingen's ‘PhD Cardiovascular Science’ doctoral programme, running since 2021, aims to integrate knowledge and techniques from all areas of natural science research, including physiology, within the context of cardiovascular science. Similarly, the University of Freiburg's Spemann Graduate School of Biology and Medicine provides a cardiovascular track as part of the PhD curriculum, with a wide range of seminars, lectures, practical courses, and soft skills training.

Targeted, subject‐specific training during the specialisation period is central to PhD research and education, and funding bodies generally expect the provision of formal training, often specified in the form of a doctoral supervision agreement or as part of a graduate training college. The DFG supports more than 200 Research Training Groups that offer structured training and qualification for PhD researchers, preparing them for the complexities of scientific and academic careers (Deutsche Forschungsgemeinschaft (2024a, 2024b)). This is probably currently the most efficient and best‐developed sector of medical scientist training activities, although these initiatives are fixed‐term (usually up to 8 years) and thereby lack sustainability.

*Path to professional independence*: The Hannover Medical School's Medical Scientist Programme, established in 2021, is one of six current EKFS‐funded schools and offers postdoctoral researchers advanced training in basic biomedical research and financial support for subject areas including cardiovascular sciences. Assistance for advanced medical scientists includes 3 years of research project funding, specialist training, mentoring, and networking opportunities. Other medical scientist programmes, such as the University of Freiburg's Hans A. Krebs Medical Scientist Programme, offer mentoring and financial support for shorter periods (e.g., €40,000 per year for 2 years) without a stated subject focus.

These programmes are probably the closest analogues to clinician scientist support structures. Together with individual fellowships of the DGK, DZHK, and DFG, they can support the early development of an independent research focus for medical scientists, allowing subsequent application for competitive national fellowships.

## APPENDIX B What can be done?

### B.1

Much remains to be done to improve the standing, training, and support available for physiologists and other medical scientists. Here we detail specific actions that are needed for medical scientists working in the field of cardiovascular research in Germany.

A primary goal is to increase awareness and representation of medical scientists generally, among students, early scientists, and professional bodies who may not be familiar with the concept, opportunities, and needs of medical scientists. Professional societies like the DGK and DZHK should play an active role in ensuring that medical scientists are recognised, supported, and represented comparably to clinician scientists. Greater awareness is needed about the unsatisfactory long‐term prospects for medical scientists in academia, the predictably negative effects that time restrictions (such as those imposed by the German *WissZeitVG*) can have on the professional development of a key category of health researchers, and the need for new ideas for training and support of medical scientists. Universities and other research institutions should work with professional societies like the DGK and DZHK to provide advice and information to scientists on topics like the *WissZeitVG*, for example, to ensure that their students and staff receive information from a reputable source and that their views and suggestions are heard. Researchers themselves can promote this through participation in early career communities (such as the Young DZHK and Young DGK) to shape the future of medical scientists. Defining a medical scientist “brand” may help improve awareness, recognition, networking, lobbying, and thus representation. In this case, the DPG has taken an important step with the introduction of the “specialist physiologist” title in Germany, providing a framework in which physiologists can demonstrate their specialist knowledge and ability to perform independent scientific research. Similar qualifications could be established elsewhere to improve the recognition and reputation of medical scientists working in other areas of research or outside Germany.

Suitable and coherent education and specialisation for medical scientists are needed throughout their progression from basic training (university degree) to specialisation (PhD research) and on toward professional independence (postdoc and principal investigator). Training and financial support should begin during or before the specialisation phase, as with the highly successful clinician scientist programmes. Distinguishing between “early” (e.g., the specialisation phase) and “advanced” stages (toward professional independence) may be useful, as needs differ in these career stages, among both clinician scientists and medical scientists. The DZHK, for instance, has funding options for both early‐stage and advanced postdocs, for which clinicians and medical scientists are equally eligible to apply.

A current challenge for medical scientists is to link training and further education to formal recognition of specialisation via a certification that would benefit eligibility for support, employability, and career progression. For physicians, certification of attendance at specific CME courses is both expected and recorded; this is not presently the case for medical scientists. A shared cardiovascular curriculum for medical scientists, developed by the relevant professional bodies, would go a long way toward providing the training that medical scientists and their field need.

Medical scientists' access to fair and adequate funding opportunities needs to be ensured throughout the formative years of their professional development. Currently, few funding programmes are tailored to support medical scientists, and most apply to post‐PhD stages. More substantial and more targeted financial support is needed for medical scientists at graduate and postgraduate levels, such as via fellowships, dedicated bridging programmes, pump‐priming support, or partial funding of salaries of medical scientists who are applying for their first grant with the DFG. Funding is particularly scarce for medical scientists‐in‐training, that is, during their PhD studies. Furthermore, the DGK and DZHK should explore supporting existing medical scientist training programmes in cardiovascular sciences, such as via funding for qualification and networking (e.g., free attendance at DGK and DZHK meetings) and travel support.

There is a need to improve the long‐term career prospects for physicians and medical scientists in general. This involves introducing junior scientists to the options available beyond academia in a more targeted manner. Professional societies are well placed to support this, for example, by ensuring that medical scientists are given broad representation among invited faculty for training and scientific events. At the same time, there is a need for concerted resistance to well meaning but ill‐considered proposals at administrative and political levels, such as the imminent tightening of legally mandated time limits for postdoctoral work. More must be done to ensure that medical scientists have a reasonable chance for a stable, long‐term career, within and beyond academia.
